# Decreasing level of resistance in invasive *Klebsiella pneumoniae* strains isolated in Marseille, January 2012–July 2015

**DOI:** 10.1186/s40064-016-2296-0

**Published:** 2016-05-17

**Authors:** Cédric Abat, Didier Raoult, Jean-Marc Rolain

**Affiliations:** URMITE UM 63 CNRS 7278 IRD 198 INSERM U1905, IHU Méditerranée Infection, Faculté de Médecine et de Pharmacie, Aix-Marseille Université, 27 boulevard Jean Moulin, 13385 Marseille Cedex 05, France

**Keywords:** Historical database, *K. pneumoniae*, Laboratory-based surveillance system, Carbapenem

## Abstract

**Background:**

*Klebsiella pneumoniae* is a Gram-negative bacterial species well known for its capacity to cause infections in humans, and to carry and spread a wide variety of resistance genes including extended-spectrum beta-lactamase genes, carbapenem resistance genes, and colistin resistance genes. Recently, our real-time laboratory-based surveillance system MARSS (the Marseille Antibiotic Resistance Surveillance System) allowed us to observe a intringing dramatic decrease in the beta-lactam resistance level of the *K. pneumoniae* strains routinely isolated from patients hospitalized in our settings since 2013. Here we study the evolution of the prevalence of *K. pneumoniae* infections in Marseille university hospitals, France, from January 2012 to July 2015, and study their antibiotic resistance profiles.

**Methods:**

We collected data referring to patients hostpitalized for *K. pneumoniae* infections in the 4 university hospitals of Marseille from January 2012 to July 2015. We then study their antibiotic resistance profiles according the clinical sites from which each strain was collected. Antibiotic consumption data from our four hospitals were also analyzed from January 2013 to July 2015.

**Results:**

Overall, 4868 patients were admitted in our settings for *K. pneumoniae* infections over the study period. Overall, 40.1, 22.3, 25.6, 0.4, 29.9, 14.8, 27.3 and 37.0 % of the strains were resistant to amoxicillin plus clavulanic acid, piperacillin-tazobactam, ceftriaxone, imipenem, ciprofloxacin, gentamicin, trimethoprim-sulfamethoxazole and furan, respectively. 447 were invasive infections. The resistance level of our invasive strains was significantly lower than that presented by 11, 7, 10 and 11 other European countries included in the 2013 European Antimicrobial Resistance Surveillance Network report for ceftriaxone, imipenem, ciprofloxacin and gentamicin, respectively, but significantly higher than that of 13, 1, 17 and 13 European countries for the same antibiotics. We also observed that the percentages of resistance of our invasive strains to three of the four antibiotics decreased over the study. In parallel, antibiotic consumption remained stable in our four hospitals from January 2013 to July 2015.

**Conclusions:**

Altogether, our results underline that automated antibiotic-susceptibility testing results-based surveillance systems are crucial to better understand the evolving epidemiology of dangerous pathogenic bacterial species, like *K. pneumoniae*, at local scales.

**Electronic supplementary material:**

The online version of this article (doi:10.1186/s40064-016-2296-0) contains supplementary material, which is available to authorized users.

## Background

*Klebsiella pneumoniae* is a non-motile, rod-shaped, Gram-negative bacterium naturally present in the environment but equally in humans, where it can colonize the nasopharynx, the skin, but equally the gastrointestinal tract (Berrazeg et al. 2013; Ramos et al. 2014). This bacterial species is well known worldwide for its capacity to cause infections in humans (mostly blood stream, urinary and respiratory tract infections), especially in hospitalized patients with impaired immune systems like diabetics and newborns (European Centre for Disease Prevention and Control 2013). Because of its capacity to survive on the skin and to spread rapidly in the hospital environment, it can be responsible for large nosocomial outbreaks transferred via the hands of hospital personnel (Ramos et al. 2014; European Centre for Disease Prevention and Control 2013).

Similar to other Enterobacteriaceae, *K. pneumoniae* has extraordinary capacities for carrying and spreading a wide variety of resistance genes including extended-spectrum beta-lactamase genes like SHV, CTX and AmpC (Harris et al. 2015), carbapenem resistance genes including NDM, KPC, IMP and VIM (Rolain et al. 2010; Nordmann and Carrer 2010), and more recently colistin resistance genes, especially *mgrB*, *pmr*A, *pmr*B, *pho*P and *pho*Q genes (Olaitan et al. 2014). Infections with multidrug-resistant *K. pneumoniae* represent real public health challenges. Numerous studies have shown that these infections increase the mortality, the cost of treatment and the hospital stays of infected patients (Schwaber and Carmeli 2007; Daroukh et al. 2014).

In order to detect and quickly fight possible hospital outbreaks due to multidrug-resistant bacterial strains belonging to 15 bacterial species of clinical interest (*Escherichia coli, Klebsiella pneumoniae*, *Proteus mirabilis*, *Enterobacter cloacae*, *Klebsiella oxytoca*, *Enterobacter aerogenes, Morganella morganii*, *Serratia marcescens, Pseudomonas aeruginosa*, *Acinetobacter baumannii*, *Streptococcus agalactiae*, *Enterococcus faecalis, Enterococcus faecium*, *Staphylococcus aureus* and *Staphylococcus epidermidis*), we decided in 2013 to implement, based on data routinely produced by the four university hospitals of Marseille, our own real-time laboratory-based surveillance system, MARSS (the Marseille Antibiotic Resistance Surveillance System) (Abat et al. 2015). This surveillance system allowed us to observe a dramatic decrease in the beta-lactam resistance level of the *K. pneumoniae* strains routinely isolated from patients hospitalized in our settings since 2013.

Here we present and study the level of resistance to antibiotics of *K. pneumoniae* strains isolated from patients admitted in our settings between January 2012 and July 2015. We then compare our results with available data.

## Methods

All the data studied herein were retrospectively collected from the laboratory management system of the four university hospitals of Marseille. These hospitals included the North (approximately 600 beds), South (900 beds), Conception (700 beds) and Timone (1500 beds) hospitals. The collected data were raw data on antibiotic susceptibility testing routinely performed on *K. pneumoniae* strains isolated from hospitalized patients between January 2012 and July 2015. All the *K. pneumoniae* strains included in the study were identified using Matrix Assisted Laser Desorption Ionisation—Time of Flight (MALDI-TOF) mass spectrometers according to the MALDI-TOF identification score previously defined and published by our laboratory (i.e. identification score ≥1.9 for good species identification) (Seng et al. 2013, 2009). *K. pneumoniae* mass spectrometry spectra currently present in our spectra database are presented in Additional file 1: Table S1.

**Fig. 1:**
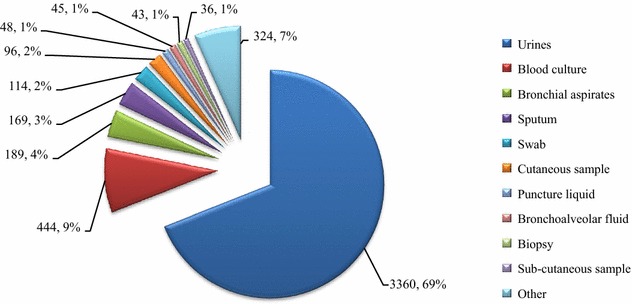
Main samples infected by our 4868 *K. pneumoniae* strains, January 2012–July 2015

Once collected, the data were processed in a Microsoft Excel database, and duplicates were removed to conserve single bacteria-patient couples to ensure the good quality of the analysis performed here. The infections were classified according to the sample from which each *K. pneumoniae* strain was isolated (Fig. 1).

Extracted data included antibiotic results for amoxicillin plus clavulanic acid, piperacillin-tazobactam, ceftriaxone, imipenem, ciprofloxacin, gentamicin, trimethoprim-sulfamethoxazole and furan. In our laboratory, antibiotic susceptibility testing is performed following the EUCAST recommendations. Therefore, all were obtained performing disk diffusion tests. Moreover, E tests of imipenem are routinely performed to validate or not possible imipenem-resistant *K. pneumoniae* resistance phenotypes. Percentages of resistance to ceftriaxone, imipenem, ciprofloxacin and gentamicin of our invasive strains (meaning our *K. pneumoniae* strains responsible for bacteremia or meningitis) were compared to those available in the 2013 European Antimicrobial Resistance Surveillance Network (EARS-Net) report (European Centre for Disease Prevention and Control 2013). These data only included one bacteremia or meningitis record per patient infected by the bacterium at the community or hospital level classified per country included in the EARS-Net report. In order to determine the part of invasive *K. pneumoniae* infections that were hospital-acquired infections, we classified them according to the delay between the date of sampling and the date of hospitalization of each patient included in this study. Thus, hospital-acquired invasive *K. pneumoniae* infections were defined as infections that occurred at least 3 days after the hospitalization of the patient in our settings.

Antimicrobial consumption data from our four hospitals for Ceftriaxone, Ciprofloxacin, Gentamicin and Imipenem were extracted then sorted per hospital in Microsoft Excel sheets. Only data from January 2013 to July 2015 were analyzed (data for 2012 were not available for analysis).

Statistical analyses were performed using the R software (Auckland, New-Zealand). We performed two-sided Pearson’s Chi Square tests. p values <0.05 were considered statistically significant.

As our *K. pneumoniae* strains were collected from patients in France during standard hospital procedures, no written consent was needed, in accordance with the ‘LOI no. 2004-800 relative à la bioéthique’ published in the *Journal Officiel de la République Française*, 6 August 2004.

## Results

4868 non-redundant patients were admitted in our settings for *K. pneumoniae* infections from January 2012 to July 2015, especially for urinary-tract infections (3360 infections, 69 % of the overall number of *K. pneumoniae* infections studied here) (Fig. 1). Overall, the number of *K. pneumoniae* infections remained stable over the years and the hospitals (Table 1). In parallel, antibiotic consumption for Ceftriaxone, Ciprofloxacin, Gentamicin and Imipenem remained stable in our four hospitals from January 2013 to July 2015 (Additional file 2: Table S2).

**Table 1:** Distribution of our 4868 *K. pneumoniae* strains per kind of infection, hospitals and years

Years	All infections						Invasive infections						*p* value^b^
	North hospital	South hospital	Timone hospital	Conception hospital	Others^a^	Total	North hospital	South hospital	Timone hospital	Conception hospital	Others	Total	
2012	362	29	285	397	102	1175	32	0	31	44	8	115	0.9
2013	409	48	353	405	86	1301	44	1	29	47	9	130	
2014	404	35	342	363	134	1278	42	2	41	39	11	135	
2015^c^	207	24	170	112	154	667	22	1	17	9	18	67	
Total	1382	136	1150	1277	476	4421	140	4	118	139	46	447	

^a^ *K. pneumoniae* infections not classifiable among the different hospitals

^b^Two-sided Pearson’s Chi Square test performed comparing the total number of *K. pneumoniae* infections identified in 2012 and 2013 to the number of *K. pneumoniae* invasive infections observed the same years. *p* value of <0.05 was considered statistically significant

^c^From January 2015 to July 2015

**Fig. 2:**
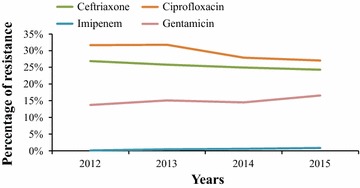
Annual resistance level of our 4868 *K. pneumoniae* strains, January 2012–July 2015

Globally, 40.1, 22.3, 25.6, 0.4, 29.9, 14.8, 27.3 and 37.0 % of the strains were resistant to amoxicillin plus clavulanic acid, piperacillin-tazobactam, ceftriaxone, imipenem, ciprofloxacin, gentamicin, trimethoprim-sulfamethoxazole and furan, respectively. The annual evolution of the percentage of resistance of all our *K. pneumoniae* strains to ceftriaxone, imipenem, ciprofloxacin and gentamicin is presented Fig. 2. Overall, these percentages of resistance did not statistically change except for imipenem, for which the resistance level of our strains significantly increased from 2012 to 2015 (*p* value: 0.02).

**Fig. 3:**
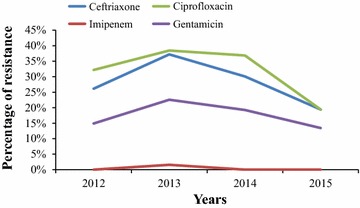
Annual resistance level of our 447 invasive *K. pneumoniae* strains, January 2012–July 2015

The comparison of the resistance level of our *K. pneumoniae* strains isolated from invasive infections with those presented by the other European countries included in the EARS-Net report is shown in Table 2. Overall, 447 invasive *K. pneumoniae* infections occurred over the study period in our settings, with a non-significant increase in the number of invasive infections between 2012 and 2013 (p value = 0.9, Table 1). 45 % of these infections (203 infections) were hospital-acquired infections. We observed that the level of resistance of our invasive strains was significantly lower than that presented by 11, 7, 10 and 11 other European countries for ceftriaxone, imipenem, ciprofloxacin and gentamicin, respectively (Table 2). On the other hand, the level of resistance of our invasive strains was significantly higher than that of 13, 1, 17 and 13 European countries for the same antibiotics (Table 2). Figure 3 shows the annual evolution of the percentage of resistance of our invasive *K. pneumoniae* strains to ceftriaxone, imipenem, ciprofloxacin and gentamicin. We observed that the percentages of resistance of our invasive *K. pneumoniae* strains to three of the four antibiotics decreased from January 2012 to July 2015 (0.7, 0.6 and 0.9-fold decrease for ceftriaxone, ciprofloxacin and gentamicin, respectively). However, none of these decreases were statistically significant between 2012 and 2015.

**Table 2:** Antibiotic resistance level of our invasive *K. pneumoniae* strains and those of 30 European countries

Country	Total number of strains ^a^	Number of strains tested and percentage of resistance per antibiotic tested																*p* value^b^			
		AMC		TZP		CRO		IMP		CIP		GM		SXT		FT		CRO	IMP	CIP	GM
		No	%	No	%	No	%	No	%	No	%	No	%	No	%	No	%				
Our study	447	127	41.7	349	29.4	440	29.5	446	0.4	445	33.5	440	1 18.2	441	31.5	441	47.2				
Austria	3315					3315	12.0	2767	0.8	3271	16.4	3315	5.8					p < 10^−3^	0.7	p < 10^−3^	p < 10^−3^
Belgium	1978					1945	14.9	1925	0.4	1978	17.7	1844	8.9					p < 10^−3^	1	p < 10^−3^	p < 10^−3^
Bulgaria	513					513	75.1	479	0.4	512	49.0	512	62.5					p < 10^−3^	1	0.002	p < 10^−3^
Croatia	1289					1288	51.3	1285	0.6	1274	43.9	1289	47.6					p < 10^−3^	1	0.01	p < 10^−3^
Cyprus	283					283	32.9	283	12.0	283	30.4	283	23.0					0.6	p < 10^−3^	0.6	0.2
Czech Republic	5240					5240	50.0	4736	0.3	5240	51.5	5218	49.3					p < 10^−3^	0.8	p < 10^−3^	p < 10^−3^
Denmark	3473					2346	10.9	2405	0.1	3376	10.1	3473	5.6					p < 10^−3^	0.4	p < 10^−3^	p < 10^−3^
Estonia	338					304	22.4	292	1.0	332	22.6	338	14.5					0.1	0.6	0.01	0.3
Finland	1883					1883	2.5	1877	0.0	1874	2.4	1815	1.6					p < 10^−3^	0.04	p < 10^−3^	p < 10^−3^
France	6845					6845	23.7	6541	0.3	6817	26.1	6287	23.1					0.03	1	0.01	0.07
Germany	2407					2407	13.8	2380	0.2	2405	14.4	2402	9.3					p < 10^−3^	0.7	p < 10^−3^	p < 10^−3^
Greece	6018					6018	73.1	5992	59.2	5911	70.3	5939	63.5					p < 10^−3^	p < 10^−3^	p < 10^−3^	p < 10^−3^
Hungary	2000					2000	44.3	1916	3.0	1964	42.8	2000	44.2					p < 10^−3^	0.004	0.02	p < 10^−3^
Iceland	99					97	6.2	97	0.0	90	2.2	99	0.0					p < 10^−3^	1	p < 10^−3^	p < 10^−3^
Ireland	1277					1264	11.2	1258	0.1	1275	9.7	1277	10.3					p < 10^−3^	0.6	p < 10^−3^	p < 10^−3^
Italy	3702					3621	50.1	3644	28.0	3556	48.8	3702	39.3					p < 10^−3^	p < 10^−3^	p < 10^−3^	p < 10^−3^
Latvia	299					299	56.9	297	0.0	292	44.9	299	47.5					p < 10^−3^	0.7	0.05	p < 10^−3^
Lithuania	549					549	55.9	391	0.0	545	49.7	549	55.6					p < 10^−3^	0.5	0.001	p < 10^−3^
Luxembourg	210					210	26.2	206	0.5	210	22.8	210	21.4					0.5	1	0.05	0.5
Malta	235					235	20.4	235	3.4	235	21.3	235	19.2					0.06	0.008	0.01	0.9
Netherlands	2711					2688	7.4	2692	0.2	2680	6.5	2711	6.8					p < 10^−3^	0.7	p < 10^−3^	p < 10^−3^
Norway	2166					2166	3.1	2159	0.2	2115	4.9	2163	2.3					p < 10^−3^	0.6	p < 10^−3^	p < 10^−3^
Poland	1363					1248	57.9	1343	0.6	1323	57.8	1363	48.9					p < 10^−3^	1	p < 10^−3^	p < 10^−3^
Portugal	2907					2888	35.4	2676	1.1	2879	35.0	2907	30.2					0.1	0.3	0.7	p < 10^−3^
Romania	358					358	64.0	341	17.0	340	17.0	339	17.0					p < 10^−3^	p < 10^−3^	0.004	p < 10^−3^
Slovakia	1333					1332	66.0	1107	2.3	1330	68.2	1333	64.5					p < 10^−3^	0.02	p < 10^−3^	p < 10^−3^
Slovenia	927					927	27.7	926	0.3	927	31.8	927	21.3					0.6	1	0.7	0.3
Spain	4700					4700	15.1	4698	0.7	4697	17.3	4700	12.4					p < 10^−3^	0.7	p < 10^−3^	0.003
Sweden	3999					3910	2.7	3999	0.0	3473	3.6	3653	2.3					p < 10^−3^	0.07	p < 10^−3^	p < 10^−3^
United Kingdom	3998					3686	10.3	3441	0.4	3944	7.1	3998	5.5					p < 10^−3^	1	p < 10^−3^	p < 10^−3^

Data from the 2013 European Antimicrobial Resistance Surveillance Network report, www.ecdc.europa.eu/en/publications/Publications/antimicrobial-resistance-surveillance-europe-2013.pdf. Invasive infections are bacteraemia or meningitis

*AMC* Amoxicillin plus clavulanic acid, *TZP* Piperacillin-tazobactam, *CRO* Ceftriaxone, *IMP* Imipenem, *CIP* Ciprofloxacin, *GM* Gentamicin, *SXT* Trimethoprim-sulphamethoxazole, *FT* Furan

^a^The number of *K. pneumoniae* strains were estimations based on data presented in the 2013 European Antimicrobial Resistance Surveillance Network report for the other European countries from 2010 to 2013. These calculations were based on the maximum of strains tested for antibiotic susceptibility testing for every year from 2010 to 2013

^b^Analyses performed using Pearson Chi Square or Fisher exact tests as appropriate (two-sided tests, p value <0.05 considered as statistically significant). Statistical tests were performed comparing the number of invsiave *K. pneumoniae* strains resistant to ceftriaxone, imipenem, ciprofloxacin and gentamicin in our study to that of the different countries included in the 2013 European Antimicrobial Resistance Surveillance Network report

## Discussion

*K. pneumoniae* is an important pathogen that can carry and spread various resistance genes in the community and at the hospital level. Indeed, from 1980 to 2000, *K. pneumoniae* strains were found to carry and disperse various resistance genes worldwide, especially through nosocomial infections, including TEM, SHV and CTX-M type extended-spectrum beta-lactamase, and AmpC cephalosporinase (Molton et al. 2013). They were also involved in the global spread of carbapenemase encoding genes, including KPC enzymes (Munoz-Price et al. 2013).

Our results allowed us to observe that the *K. pneumoniae* strains responsible for invasive infections in our hospitals from January 2012 to July 2015 were significantly more resistant to antibiotics (excluding imipenem) than most of those responsible for invasive infections in the other European countries included in the 2013 EARS-Net report (European Centre for Disease Prevention and Control 2013) (Table 2). However, interestingly, our results also allowed us to identify that over the study period, the annual resistance level of our invasive *K. pneumoniae* strains to the four antibiotics of interest globally decreased (Fig. 3). This is surprising, especially when the 2013 EARS-Net report observed the opposite trends since 2010 in most of the European countries, including France (European Centre for Disease Prevention and Control 2013). These observations could be explained by local successive emergence-replacement events of more or less resistant *K. pneumoniae* clones expressing genes making them particularly adapted to our environment. A similar phenomenon was observed and described by Ramos et al. in 2014. Indeed, after analyzing the genomic content of a specific *Klebsiella pneumoniae* carbapenemase (KPC)-2-producing *K. pneumoniae* clone called Kp 13 responsible for a large nosocomial outbreak in a teaching hospital located in the south of Brazil (Ramos et al. 2014), the authors concluded that the genes harbored by this *K. pneumoniae* clone might explain its ability to rapidly spread at the hospital level.

The peak of antibiotic resistance especially observed in invasive *K. pneumoniae* strains isolated in 2013 in our hospitals (Fig. 3) can be explained by successive oxacillinase-48 carbapenemase–producing *K. pneumoniae* real nosocomial outbreaks identified by MARSS involving patients especially hospitalized in our intensive care units the same year (Abat et al. 2015). Although we continue to isolate few OXA-48 producing *K. pneumoniae* strains, the measures taken in our hospitals to fight this threat led to the dramatic decrease in the number of isolation of OXA-48 producing *K. pneumoniae* strains, which can possibly explain the lower antibiotic resistance level of the *K. pneumoniae* strains isolated from 2014 to 2015 in our settings (Figs. 2, 3).

Our results finally underlined that only a few numbers of all our *K. pneumoniae* strains (20 strains, 0.4 %) were resistant to imipenem (Table 2; Fig. 3). This is good news considering the fact that our region is closed to Italy and Greece, two countries where KPC-producing *K. pneumoniae* strains have become endemic since 2008 and 2007, respectively (Munoz-Price et al. 2013). Moreover, a recent study observed that molecules classified as ‘old antibiotics’ present good in vitro activity against highly resistant Gram-negative bacteria, including imipenem-resistant *K. pneumoniae* (Dubourg et al. 2015).

The fact that we did not perform a further genomic analysis of local *K. pneumoniae* strains responsible for invasive infections represents a major limitation of our study. We believe that such analyses should be performed in the future to better characterize the specific *K. pneumoniae* epidemiology of our region. Another major limitation consists in the fact that our definition of hospital-acquired infections did not take into account the possible movements of patients between different hospitals, which can introduce bias in our calculations of the number of hospital and/or community-acquired infections.

In conclusion, our results allowed us to conclude that automated laboratory-based surveillance systems implemented for the monitoring of antibiotic-susceptibility testing results of *K. pneumoniae* strains isolated from hospitalized patients are crucial to quickly identify resistant clone outbreaks (Abat et al. 2015), but equally to better understand the evolving epidemiology of this dangerous pathogenic bacterial species at local scales.

## Additional files

10.1186/s40064-016-2296-0 List of the 33 *K. pneumoniae* mass spectrometry spectra included in our MALDI-TOF spectra database.
10.1186/s40064-016-2296-0 Antimicrobial consumption data per hospital for Ceftriaxone, Ciprofloxacin, Gentamicin and Imipenem from January 2013 to July 2015.

## Abbreviations

MARSS: the Marseille Antibiotic Resistance Surveillance System
MALDI-TOF: Matrix Assisted Laser Desorption Ionisation—Time of Flight
EARS-Net: the European Antimicrobial Resistance Surveillance Network
KPC: *Klebsiella pneumoniae* carbapenemase
OXA: oxacillinase
