# Supplementary material for: Decreasing level of resistance in invasive Klebsiella pneumoniae strains isolated in Marseille, January 2012–July 2015
Source: Springerplus. 2016 May 17;5:631. doi: 10.1186/s40064-016-2296-0 (PMC4870489; doi:10.1186/s40064-016-2296-0)
Supplement: Supplementary file 1 — 10.1186/s40064-016-2296-0 List of the 33 K. pneumoniae mass spectrometry spectra included in our MALDI-TOF spectra database. [file 40064_2016_2296_MOESM1_ESM.docx]

**Additional file 1: Table S1.** List of the 33 *K. pneumoniae* mass spectrometry spectra included in our MALDI-TOF spectra database.

| **Name of the spectra** | **Origin of the spectra** |
| --- | --- |
| Klebsiella pneumomiae CSURP348 | Collection de Souche de l’Unité des Rickettsies |
| Klebsiella pneumoniae CSURP130 | Collection de Souche de l’Unité des Rickettsies |
| Klebsiella pneumoniae CSURP131 | Collection de Souche de l’Unité des Rickettsies |
| Klebsiella pneumoniae CSURP215 | Collection de Souche de l’Unité des Rickettsies |
| Klebsiella pneumoniae CSURP217 | Collection de Souche de l’Unité des Rickettsies |
| Klebsiella pneumoniae CSURP218 | Collection de Souche de l’Unité des Rickettsies |
| Klebsiella pneumoniae CSURP219 | Collection de Souche de l’Unité des Rickettsies |
| Klebsiella pneumoniae CSURP220 | Collection de Souche de l’Unité des Rickettsies |
| Klebsiella pneumoniae CSURP2205 | Collection de Souche de l’Unité des Rickettsies |
| Klebsiella pneumoniae CSURP221 | Collection de Souche de l’Unité des Rickettsies |
| Klebsiella pneumoniae CSURP224 | Collection de Souche de l’Unité des Rickettsies |
| Klebsiella pneumoniae CSURP225 | Collection de Souche de l’Unité des Rickettsies |
| Klebsiella pneumoniae CSURP226 | Collection de Souche de l’Unité des Rickettsies |
| Klebsiella pneumoniae CSURP227 | Collection de Souche de l’Unité des Rickettsies |
| Klebsiella pneumoniae CSURP228 | Collection de Souche de l’Unité des Rickettsies |
| Klebsiella pneumoniae CSURP229 | Collection de Souche de l’Unité des Rickettsies |
| Klebsiella pneumoniae CSURP230 | Collection de Souche de l’Unité des Rickettsies |
| Klebsiella pneumoniae subsp ozaenae CSURP249 | Collection de Souche de l’Unité des Rickettsies |
| Klebsiella pneumoniae subsp pneumoniae CSURP250 | Collection de Souche de l’Unité des Rickettsies |
| Klebsiella pneumoniae subsp. rhinoscleromatis CSURP248 | Collection de Souche de l’Unité des Rickettsies |
| Klebsiella pneumoniae ssp ozaenae CCM 5792T CCM | Czech Collection of Microorganisms |
| Klebsiella pneumoniae ssp rhinoscleromatis CCM 5791T CCM | [Czech Collection of Microorganisms](http://www.sci.muni.cz/ccm/) |
| Klebsiella pneumoniae ssp ozaenae DSM 16358T DSM | Deutsche Sammlung von Mikroorganismen und Zellkulturen |
| Klebsiella pneumoniae ssp ozaenae DSM 16358T HAM | Deutsche Sammlung von Mikroorganismen und Zellkulturen |
| Klebsiella pneumoniae ssp pneumoniae DSM 30104T HAM | Deutsche Sammlung von Mikroorganismen und Zellkulturen |
| Klebsiella pneumoniae ssp pneumoniae DSM 30104T_QC DSM | Deutsche Sammlung von Mikroorganismen und Zellkulturen |
| Klebsiella pneumoniae ssp rhinoscleromatis DSM 16231T HAM | Deutsche Sammlung von Mikroorganismen und Zellkulturen |
| Klebsiella pneumoniae 10088251 | Clinical laboratory of the Timone hospital |
| Klebsiella pneumoniae 37585 PFM | Clinical laboratory of the Timone hospital |
| Klebsiella pneumoniae 37595 PFM | Clinical laboratory of the Timone hospital |
| Klebsiella pneumoniae 37924 PFM | Clinical laboratory of the Timone hospital |
| Klebsiella pneumoniae RV_BA_03_B LBK | Clinical laboratory of the Timone hospital |
| Klebsiella pneumoniae ssp pneumoniae 9295_1 CHB | Clinical laboratory of the Timone hospital |
