# Supplementary material for: Decreasing level of resistance in invasive Klebsiella pneumoniae strains isolated in Marseille, January 2012–July 2015
Source: Springerplus. 2016 May 17;5:631. doi: 10.1186/s40064-016-2296-0 (PMC4870489; doi:10.1186/s40064-016-2296-0)
Supplement: Supplementary file 2 — 10.1186/s40064-016-2296-0 Antimicrobial consumption data per hospital for Ceftriaxone, Ciprofloxacin, Gentamicin and Imipenem from January 2013 to July 2015. [file 40064_2016_2296_MOESM2_ESM.docx]

**Additional file 2: Table S2.** Antimicrobial consumption data per hospital for Ceftriaxone, Ciprofloxacin, Gentamicin and Imipenem from January 2013 to July 2015.

| **Years** | **Ceftriaxone*** | | | | | **Ciprofloxacin** | | | | | **Gentamicin** | | | | | **Imipenem** | | | | |
| --- | --- | --- | --- | --- | --- | --- | --- | --- | --- | --- | --- | --- | --- | --- | --- | --- | --- | --- | --- | --- |
|  | **H1** | **H2** | **H3** | **H4** | **Total** | **H1** | **H2** | **H3** | **H4** | **Total** | **H1** | **H2** | **H3** | **H4** | **Total** | **H1** | **H2** | **H3** | **H4** | **Total** |
| **2013** | 8299 | 10591 | 752 | 13367 | **33008** | 17129 | 16794 | 2775 | 10344 | **47040** | 3078 | 2717 | 340 | 1668 | **7804** | 6715 | 5614 | 489 | 3248 | **16065** |
| **2014** | 10735 | 11033 | 675 | 11864 | **34306** | 17438 | 15961 | 2775 | 7429 | **43603** | 3176 | 2384 | 297 | 1941 | **7798** | 6444 | 4945 | 504 | 2655 | **14547** |
| **2015**** | 10761 | 6427 | 491 | 4234 | **21913** | 14866 | 8811 | 1261 | 2914 | **27851** | 2566 | 1338 | 87 | 722 | **4713** | 4514 | 2573 | 183 | 1069 | **8339** |

H1: Timone hospital

H2: North hospital

H3: South hospital

H4: Conception hospital

*: Rounded values expressed in Daily Defined Dose (DDD)

**: From January 2015 to July 2015
